# Whole genome resequencing data sets of different species from *Pistacia* genus

**DOI:** 10.1186/s13104-021-05702-9

**Published:** 2021-07-27

**Authors:** Ali Tajabadi, Ali Esmailizadeh

**Affiliations:** 1 Pistachio Research Center, Horticultural Sciences Research Institute, Agricultural Research, Education and Extension Organization (AREEO), Rafsanjan, Iran; 2 Department of Animal Science, Faculty of Agriculture, Shahid Bahonar University of Kerman, PB 76169-133, Kerman, Iran; 3 State Key Laboratory of Genetic Resources and Evolution, Kunming Institute of Zoology, Chinese Academy of Sciences, No. 32 Jiaochang Donglu, Kunming, 650223 Yunnan China

**Keywords:** Cultivars, Pistachio, Genomes, Whole-genome resequencing

## Abstract

**Objectives:**

*Pistacia* genus belongs to the flowering plants in the cashew family and contains at least 11 species. The whole-genome resequencing data of different species from *Pistacia* genus are described herein. The data reported here will be useful for better understand the adaptive evolution, demographic history, genetic diversity, population structure, and domestication of pistachio.

**Data description:**

Genomic DNA was isolated from fresh leaves and used to construct libraries with insert size of 350 bp. Sequence libraries were made and sequenced on the Illumina Hiseq 4000 platform to produce 150 bp paired-end reads. A total number of 4,851,118,730 billion reads (ranging from 33,305,900 to 34,990,618 reads per sample) were created across all samples. We produced a total of 727.67 Gbp data which have been deposited in the Genome Sequence Archive (GSA) database with the Accession of CRA000978. All of the data are also available as the sequence read archive (SRA) format in the National Center for Biotechnology Information (NCBI) with identifier of SRP189222, mirroring our deposited data in GSA.

## Objective

*Pistacia* genus belongs to the flowering plants in the Anacardiaceae family. Other plants in the Anacardiaceae or the cashew family include poison oak, mango, poison ivy, sumac, and pepper tree [1]. The *Pistacia* covers at least eleven species and is estimated to be approximately 80 million years old [2]. Pistachio has a long history of plantation (3000–4000 years) in Iran and is native to the arid zones of Central Asia [3]. The Romans at the beginning of the Christian era introduced this plant into Mediterranean Europe [3] and its cultivation extended westward from its center of origin to Italy, Spain, and other Mediterranean regions of Southern Europe, North Africa, and the Middle East, as well as to China and to the United States and Australia [4, 5]. The worldwide production of pistachios was about 1.4 million tonnes in 2018, with Iran and the United States together accounting for 72% of the total as leading producers [6]. Pistachio plants have a juvenile period of about 5–10 years. The most economically important species is *P. vera* which is the only cultivated species from the *Pistacia* genus [7]. The other species of this genus are forest trees and have edible seeds and can be used as rootstock seed sources for cultivated *P. vera* [1, 8]. Also, plant materials such as leaf, seed, flower, and resins derived from the stem of some species from the *Pistacia* genus have pharmacological properties such as antioxidant, anti-inflammatory and antimicrobial activities [9–11].

This study provides whole-genome resequencing data of different species from *Pistacia* genus (Table 1). These genome sequences data will be useful for comparative population genomics and to better understand the demographic history and adaptive evolution of pistachio. We used these data for providing insights into pistachio genetic diversity, population structure, and domestication [12].

**Table 1:** Overview of data files/data sets

Label	Name of data file/data set	File types (file extension)	Data repository and identifier (DOI or Accession Number)
Data set 1	Genome and transcriptome of pistachio	Fastq (fq.gz)	Sequence Read Archive https://www.ncbi.nlm.nih.gov/sra/SRP189222 [14]
Data set 2	CRR030744: Genome of *P. vera L.* (Ohadi Cultivar)	Fastq (fq.gz)	NGDC, Genome Sequence Archive https://bigd.big.ac.cn/gsa/browse/CRA000978/CRR030744 [15]
Data set 3	CRR030745: Kaleh Ghochi Cultivar	Fastq (fq.gz)	NGDC, Genome Sequence Archive https://bigd.big.ac.cn/gsa/browse/CRA000978/CRR030745 [16]
Data set 4	CRR030764:Akbari Cultivar	Fastq (fq.gz)	NGDC, Genome Sequence Archive https://bigd.big.ac.cn/gsa/browse/CRA000978/CRR030764 [17]
Data set 5	CRR030765: Ahmad Aqaei Cultivar	Fastq (fq.gz)	NGDC, Genome Sequence Archive https://bigd.big.ac.cn/gsa/browse/CRA000978/CRR030765 [18]
Data set 6	CRR030752: Badami Zarand Cultivar	Fastq (fq.gz)	NGDC, Genome Sequence Archive https://bigd.big.ac.cn/gsa/browse/CRA000978/CRR030752 [19]
Data set 7	CRR030840: *Pistacia integerrima*	Fastq (fq.gz)	NGDC, Genome Sequence Archive https://bigd.big.ac.cn/gsa/browse/CRA000978/CRR030840 [20]
Data set 8	CRR030854: *Pistacia khinjuk*	Fastq (fq.gz)	NGDC, Genome Sequence Archive https://bigd.big.ac.cn/gsa/browse/CRA000978/CRR030854 [21]
Data set 9	CRR030871: *Pistacia terebinthus* subsp. palaestina	Fastq (fq.gz)	NGDC, Genome Sequence Archive https://bigd.big.ac.cn/gsa/browse/CRA000978/CRR030871 [22]
Data set 10	CRR030866: *Pistacia atlantica* subsp. mutica	Fastq (fq.gz)	NGDC, Genome Sequence Archive https://bigd.big.ac.cn/gsa/browse/CRA000978/CRR030866 [23]
Data set 11	CRR030873: *Pistacia vera* (Pistachio wild type, Sarakhs)	Fastq (fq.gz)	NGDC, Genome Sequence Archive https://bigd.big.ac.cn/gsa/browse/CRA000978/CRR030873 [24]

## Data description

The materials used for DNA extraction were fresh leaves collected from the germplasm collections of the Pistachio Research Institute in Rafsanjan, Iran; the pistachio germplasm of Ardakan, Iran. Leaf tissues were harvested during the 2015–2017 period and were stored at − 80 °C at the Shahid Bahonar University of Kerman, Iran, until subjected to DNA extraction. Extraction of the total genomic DNA from the fresh leaves was conducted using hexadecyl trimethyl ammonium bromide (CTAB) protocol with some modifications. NanoDrop spectrophotometer and 1% agarose gel electrophoresis were used to assess the quantity and quality of the extracted DNA, looking for a 260/280 absorbance ratio of 1.8–2.0, a single absorbance peak at 260 nm, and no evidence of significant band shearing or contamination. The isolated DNA was dissolved in 20 μl TE buffer and kept at − 20 °C for subsequent analyses. A total of 10 μg of the extracted DNA was used to construct libraries with an average insert size of 350 bp. Illumina library preparation pipeline was used as guideline for constructing the sequence libraries. The sequence libraries were sequenced on the Illumina Hiseq 4000 platform to create 150 bp paired-end reads.

The pistachio descriptor [13] was used as a guideline to measure the pistachio fruit size-related traits. The following phenotypes were recorded: fresh fruit weight with green skin (g), dried pistachio fruit weight (g), dried pistachio fruit length (mm), dried pistachio fruit diameter (mm), dried pistachio fruit width (mm), dried pistachio fruit and kernel shape, dried kernel weight (g), kernel diameter (mm), kernel width (mm), kernel length (mm).

We resequenced a total of 107 genomes from *P. vera* (93 cultivars and 14 genomes of wild pistachio) to an average depth of 6–8X. In addition, we resequenced 35 genomes from different close species, including *P. palaestina* (n = 5), *P. mutica* (n = 13), *P. khinjuk* (n = 14), and *P. integerrima* (n = 4) (Table 1). A total number of 4,851,118,730 billion reads (ranging from 33,305,900 to 34,990,618 reads per sample) were created across all samples. We produced a total of 727.67 Gbp data (The SRA data size of 303.14 GBytes).

We processed the data and conducted several analyses [12]. The quality of the raw sequence reads was assessed using FastQC (http://www.bioinformatics.babraham.ac.uk/projects/fastqc/), and the reads were mapped to the pistachio reference genome (version 1) applying BWA-MEM (http://bio-bwa.sourceforge.net/). Sorting and duplicate marking of the bam format files were conducted by Picards tools 1.56 (http://picard.sourceforge.net) and SNPs calling was performed by using Genome Analysis Toolkit (GATK) (https://gatk.broadinstitute.org/hc/en-us). A total of 14,767,700 single-base variants (SNPs) were called [12]. The five different species, i.e., *P. vera*, *P. palaestina*, *P. mutica*, *P. khinjuk*, and *P. integerrima* were clearly separated following phylogenetic analyses using the maximum likelihood and neighbor joining methods [12].

## Limitations

No genome sequence from the male pistachio plants was created in our study and this may limit some analyses related to the sex-specific traits. The geographical coverage of *P. vera* was limited to the main center of pistachio production, Iran, and the data may not be sufficient for gene flow, migration, and study on the domestication origin of pistachio. In addition, we produced the short-reads with a mean depth of 6–8X which is a medium depth and it might not be suitable for some genomic analyses.

## Data Availability

All sequence data reported here have been deposited in NGDC, GSA database (https://bigd.big.ac.cn/gsa/) under the Accession Number of CRA000978. Please see Table 1 and the references [14–24] for details and links to the data. In addition, the sequence data described in this Data note can be freely and openly accessed as the sequence read archive (SRA) format from the NCBI database (https://www.ncbi.nlm.nih.gov/sra/SRP189222). The SRA data mirror our deposited data in GSA. However, Pistachio Research Center (Rafsanjan, Iran), maintains confidentiality of information regarding the phenotypic data. Information on these phenotypic data are available upon request from the corresponding author.

## Abbreviations

GSA: Genome Sequence Archive
NCBI: National Center for Biotechnology Information
NGDC: National Genomics Data Center
SIFT: Sorting intolerant from tolerant
masl: Meters above sea level
CTAB: Cetyl trimethylammonium bromide
DNA: Deoxyribonucleic acid
TE: Tris, Ethylenediaminetetraacetic acid
SNPs: Single nucleotide polymorphisms
NGS: Next-generation sequencing
bp: Base pair
