# Correction to: Whole genome resequencing data sets of different species from *Pistacia* genus

**DOI:** 10.1186/s13104-021-05802-6

**Published:** 2021-10-20

**Authors:** Ali Tajabadipour, Ali Esmailizadeh

**Affiliations:** 1 Pistachio Research Center, Horticultural Sciences Research Institute, Agricultural Research, Education and Extension Organization (AREEO), Rafsanjan, Iran; 2 Department of Animal Science, Faculty of Agriculture, Shahid Bahonar University of Kerman, PB 76169-133, Kerman, Iran; 3 State Key Laboratory of Genetic Resources and Evolution, Kunming Institute of Zoology, Chinese Academy of Sciences, No. 32 Jiaochang Donglu, Kunming, 650223 Yunnan China

## Correction to: BMC Res Notes (2021) 14:290 https://doi.org/10.1186/s13104-021-05702-9

Following the publication of the original article [1] the authors informed us that the first author’s surname “Tajabadi” was incorrect. The correct surname is “Tajabadipour”.

The correct author name is included in the author and reference lists of this Correction, and has already been updated in the original article.
